# Surgical gestures can be used to assess surgical competence in robot-assisted surgery: A validity investigating study of simulated RARP

**DOI:** 10.1007/s11701-023-01807-4

**Published:** 2024-01-20

**Authors:** Rikke Groth Olsen, Morten Bo Søndergaard Svendsen, Martin G. Tolsgaard, Lars Konge, Andreas Røder, Flemming Bjerrum

**Affiliations:** 1 Copenhagen Academy for Medical Education and Simulation (CAMES), Center for HR & Education, The Capital Region of Denmark, Ryesgade 53B, 2100 Copenhagen, Denmark; 2 Department of Urology, Copenhagen Prostate Cancer Center, Copenhagen University Hospital - Rigshospitalet, Copenhagen, Denmark; 3 Faculty of Health and Medical Sciences, University of Copenhagen, Copenhagen, Denmark; 4 Department of Computer Science, University of Copenhagen, Copenhagen, Denmark; 5 Department of Gastrointestinal and Hepatic Diseases, Copenhagen University Hospital - Herlev and Gentofte, Herlev, Denmark

**Keywords:** Surgical gestures, Assessment, Robotic surgery, Simulation, Artificial intelligence

## Abstract

To collect validity evidence for the assessment of surgical competence through the classification of general surgical gestures for a simulated robot-assisted radical prostatectomy (RARP). We used 165 video recordings of novice and experienced RARP surgeons performing three parts of the RARP procedure on the RobotiX Mentor. We annotated the surgical tasks with different *surgical gestures*: dissection, hemostatic control, application of clips, needle handling, and suturing. The gestures were analyzed using idle time (periods with minimal instrument movements) and active time (whenever a surgical gesture was annotated). The distribution of surgical gestures was described using a one-dimensional heat map, *snail tracks.* All surgeons had a similar percentage of idle time but novices had longer phases of idle time (mean time: 21 vs. 15 s, *p* < 0.001). Novices used a higher total number of surgical gestures (number of phases: 45 vs. 35, *p* < 0.001) and each phase was longer compared with those of the experienced surgeons (mean time: 10 vs. 8 s, *p* < 0.001). There was a different pattern of gestures between novices and experienced surgeons as seen by a different distribution of the phases. General surgical gestures can be used to assess surgical competence in simulated RARP and can be displayed as a visual tool to show how performance is improving. The established pass/fail level may be used to ensure the competence of the residents before proceeding with supervised real-life surgery. The next step is to investigate if the developed tool can optimize automated feedback during simulator training.

**Supplementary Information:**

The online version contains supplementary material available at 10.1007/s11701-023-01807-4.

## Introduction

The surgeon’s experience affects the patient outcome which requires them to improve and maintain their performance throughout their careers [1, 2]. Earlier the Halstedian methodology of *‘see one, do one, teach one* was used to train surgeons but this learning and assessment technique is biased [3]. Therefore, other assessment tools and training modalities were created to overcome this challenge [4, 5]. Virtual reality (VR) simulators can be an effective training alternative to traditional intraoperative learning. VR simulators improve performance and allow surgeons to practice their skills in risk-free environments [6, 7]. The RobotiX VR-simulator can be used for training and assessment of skills for the standardized robot-assisted radical prostatectomy (RARP) [8]. The technical skills of the procedure can be assessed with assessment tools or automated performance metrics which can give feedback on instrument kinematics e.g., camera movement, instrument path length, etc. [9, 10]. These tools can be used to assess surgical skills, however, often cumulative values are used which makes it difficult for trainees to designate specific areas in the surgical procedure where they can improve [11, 12].

A novel approach to assess surgical skills could be to use surgical gestures. S*urgical gestures* are used to deconstruct and classify surgical performance into smaller components, such as ‘needle handling’, ‘cold cut’, ‘spread’, etc., through video annotation [11, 13]. The surgical performance is analyzed using surgical gestures and the interpretation of these can be used to provide feedback to surgeons and to help them improve their surgical skills [3, 14]. Former studies have used a detailed classification of surgical gestures by deconstructing suturing or dissection into multiple gestures for each action [11, 14–18]. This approach makes it more complicated and time-consuming to annotate the surgical procedure. We wanted to examine if a reduction in the number of different types of surgical gestures could be used for the assessment of surgical skills. This could potentially reduce the data load and the time spent on annotating the surgical gestures while maintaining a proficient assessment of skills.

The objective of this study was to assess surgical competence through the classification of general surgical gestures for a simulated RARP.

## Materials and methods

Messick’s framework (content, response process, internal structure, relationship with other variables, and consequences) was used to evaluate the validity evidence.

We used video recordings of three simulator modules on the RobotiX Mentor (formerly Simbionix, Surgical Science, Sweden): *bladder neck dissection, neurovascular-bundle dissection,* and *urethrovesical anastomosis* from a previous study [8]. They were performed by 11 novices (assisted to a minimum of one RARP, but no other experience with robotic surgery) and 9 experienced surgeons (performed >50 RARP). 10 of the novice surgeons and 6 of the experienced surgeons completed the test by performing each simulator module three times. Ninety-five videos recorded of novices and 66 videos recorded of experienced RARP surgeons were annotated using surgical gestures. The primary assessor (RGO) annotated all videos manually by observing five different gestures during dissection or suturing. Gestures during dissection were regular dissection (sharp and blunt dissection), hemostatic control, and application of clips. Gestures during suturing were needle handling (needle handled by any instruments and not in contact with the tissue) and suturing (whenever the needle was in contact with tissue) (examples are shown in Supplementary video 1). All three robotic arms were included when annotating the different surgical gestures. The surgical gestures were annotated using the event-logging software *Behavioral Observation Research Interactive Software (BORIS, version 8.19.4, Torino,* Italy, http://www.boris.unito.it) [19]. All data collection and video annotation were standardized to minimize threats to validity (response process).

## Data analysis

**Fig. 1:**
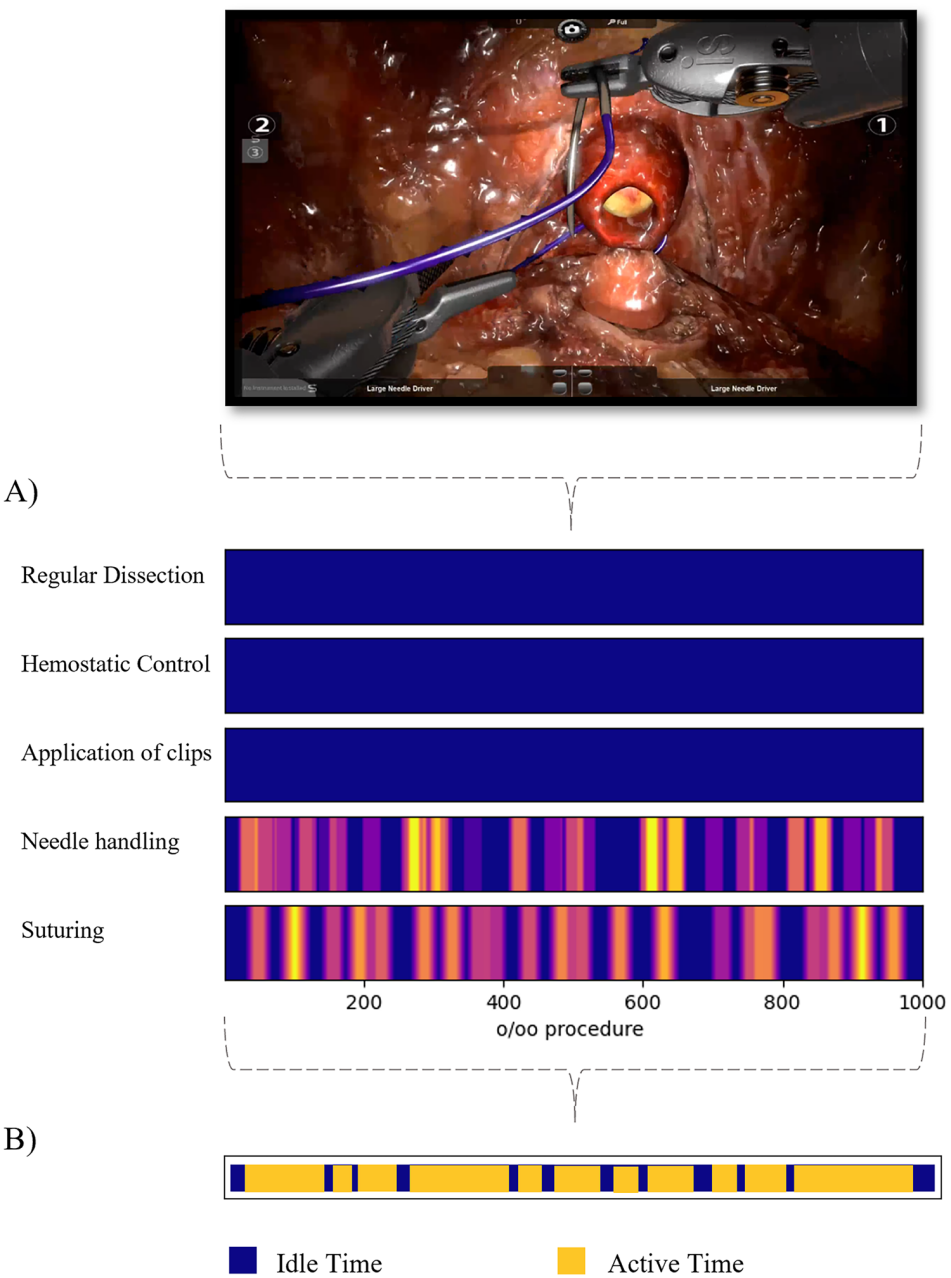
An example of annotating the surgical part-procedure with surgical gestures and transforming these into idle time and active time. (**A**) Video annotation of surgical gestures. (**B**) Transforming surgical gestures into idle time and active time

We examined the pattern of gestures to see if any surgical behavior set the two groups apart. We examined the differences using idle time and active time (Fig. 1). Idle time was defined as ‘time periods when instrument movements/interactions were minimal’ [20]. This was noted as the time between two annotated phases, where no annotation was made. Idle time was measured as the percentage of idle time in the total procedure and the mean duration of each phase of idle time. Active time was the length of each of the annotated phases and it was registered how many phases the surgeon used. Active time was measured as the total number of and mean duration of phases of surgical gestures.

All analyses were based on each participant’s average score of their repetitions. The analyses were performed for each part-procedure and as an overall total for the three part-procedures by a participant. For *internal structure*, intra-class correlation coefficients (ICC) (two-way random effects, absolute agreement, multiple measurements [21]) were calculated for the total score of each module over the three repetitions. We compared the two groups using an independent samples t-test with the statistical significance level set at 0.05 (5%) for the *relationship with other variables*. To explore the *consequences* of using surgical gestures to assess competence, we calculated a pass/fail level using the contrasting groups’ method and reported how many novices passed (i.e., false positives) and how many experienced failed (i.e., false negatives) [22]. Only participants who completed all part-procedure thrice were included in the calculated pass/fail level. The total score of the three repetitions and the average total scores of the three modules of the three repetitions were used for this analysis. The total times of each of the surgical gestures were transformed into z-scores to show how many standard deviations each score deviated from the mean score of the experienced RARP surgeons. The total time of each surgical gesture for each module and repetition was transformed into z-scores and an average z-score was calculated for each participant.

The distribution of the surgical gestures throughout the procedure was visually presented by a one-dimensional heat map, *snail tracks,* using the Python programming language (version 3.10.10, Python Software Foundation, Amsterdam, Netherlands, https://www.python.org/).

We performed a dimensional reduction using Principal Components Analysis (PCA) to create a visible feedback tool for the progression of the surgeons over repeated training tasks. PCA is an unsupervised machine learning technique for extracting relevant variables in a large dataset and condensing them into a few parameters, *principal components*. This reduces the dimensionality of the data while retaining the variation in the data set. The parameters can be plotted to create a visual representation if the samples show similarities or are grouped [23, 24]. A subsequent analysis of distance to target and magnitudes was created in these principal components. Magnitude is the length of progress between two performed procedures. For comparison of the progress between novice and experienced surgeons, we ran an independent t-test between the two groups with the level of statistical significance set at 0.05 (5%).

## Ethics

Approval by The Danish Data Protection Agency was secured (P-2020-701). The study was exempt from ethical approval by the Danish National Ethics Committee (ID: H-19016423). All videos were pseudo-anonymized with a randomly allocated identification ID before statistical analysis.

## Results

The intra-class correlation coefficients (*internal structure*) were high for all three modules (*bladder neck dissection*: ICC 0.97, 95% CI (0.89;0.99); *neurovascular-bundle dissection*: ICC 0.92, 95% CI (0.81;0.97); *urethrovesical anastomosis*: ICC 0.98, 95% CI (0.96–0.99)). When examining the *relationship with other variables*, we found that novices spend more time on the total procedure (combined of all three part-procedures) than experienced surgeons (mean time: 1150 vs. 675 s, *p* < 0.001) (Table 1). Novices and experienced surgeons have the same percentage-wise idle time in the procedures but each *idle time phase* is longer for the novices (Table 1). Furthermore, novices have a higher total number of active surgical phases throughout the procedures, but the phases are of the same length as experienced surgeons. As a result, the novices’ total surgical time is longer. For bladder-neck dissection, only the total procedure length (*p* = 0.01) and the number of phases (*p* = 0.003) were significantly longer for novices than experienced surgeons, not the idle time or mean active time.

**Table 1:** The total procedure length, idle time, and active time (in seconds) for novices and experienced surgeons

	Total procedure length	Idle time		Active time	
	Mean (SD)	Mean (SD)	%	Mean (SD)	*N*
*Total procedure*					
Novices	1150 (560)	21 (8)	61.6	10 (3)	45
Experienced surgeons	675 (365)	15 (5)	62.4	8 (3)	35
*p value*	<0.001*	<0.001*	0.78	<0.001*	<0.001*
*Bladder-neck dissection*					
Novices	713 (328)	15 (5)	40	10 (3)	39
Experienced surgeons	487 (262)	13 (5)	45	10 (3)	26
* p value*	0.01*	0.38	0.11	0.56	0.003*
*Neurovascular-bundle dissection*					
Novices	1274 (546)	20 (5)	64	9 (3)	47
Experienced surgeons	620 (258)	14 (5)	59	8 (2)	27
* p value*	<0.001*	<0.001*	0.06	0.49	<0.001*
*Urethrovesical anastomosis*					
Novices	1498 (458)	29 (6)	83	12 (2)	48
Experienced surgeons	918 (409)	16 (4)	84	7 (2)	51
* p value*	<0.001*	<0.001*	0.26	<0.001*	0.40

*Significance level of *p* < 0.05

**Fig. 2:**
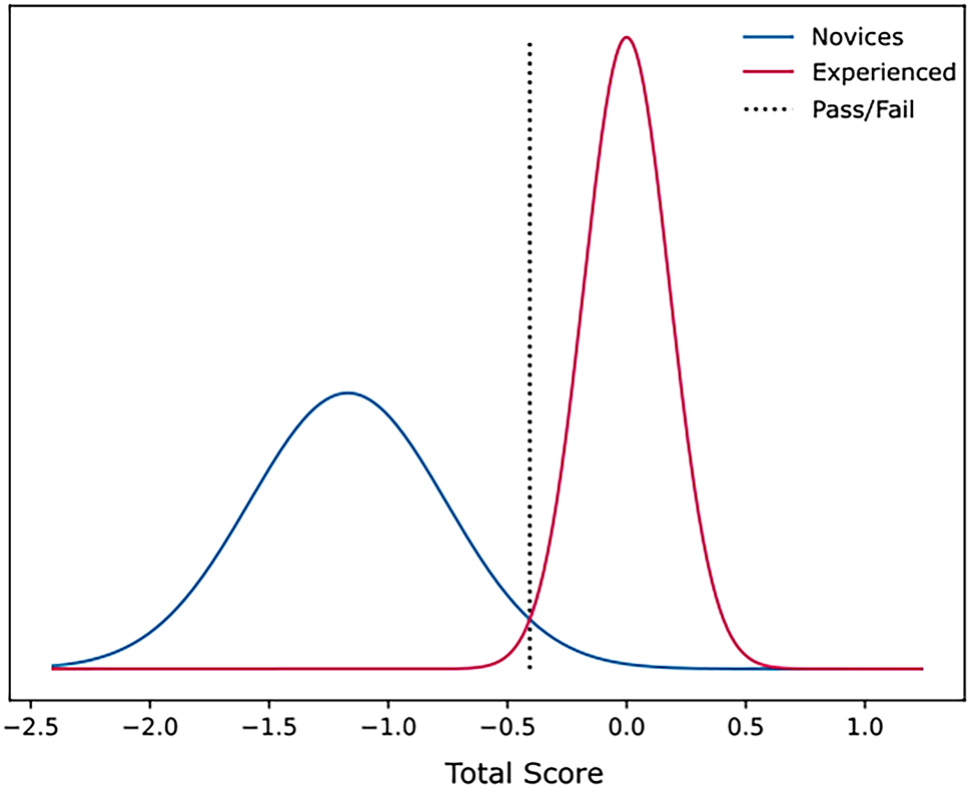
Pass/fail level between novices and experienced surgeons. All novices failed the test whereas all experienced surgeons passed

The pass/fail level (*consequences*) was identified at -0.4 standard deviations which can fully discriminate between novice and experienced surgeons (Fig. 2). Both the sensitivity and specificity were 100% as all novices failed the test whereas all experienced surgeons passed.

**Fig. 3:**
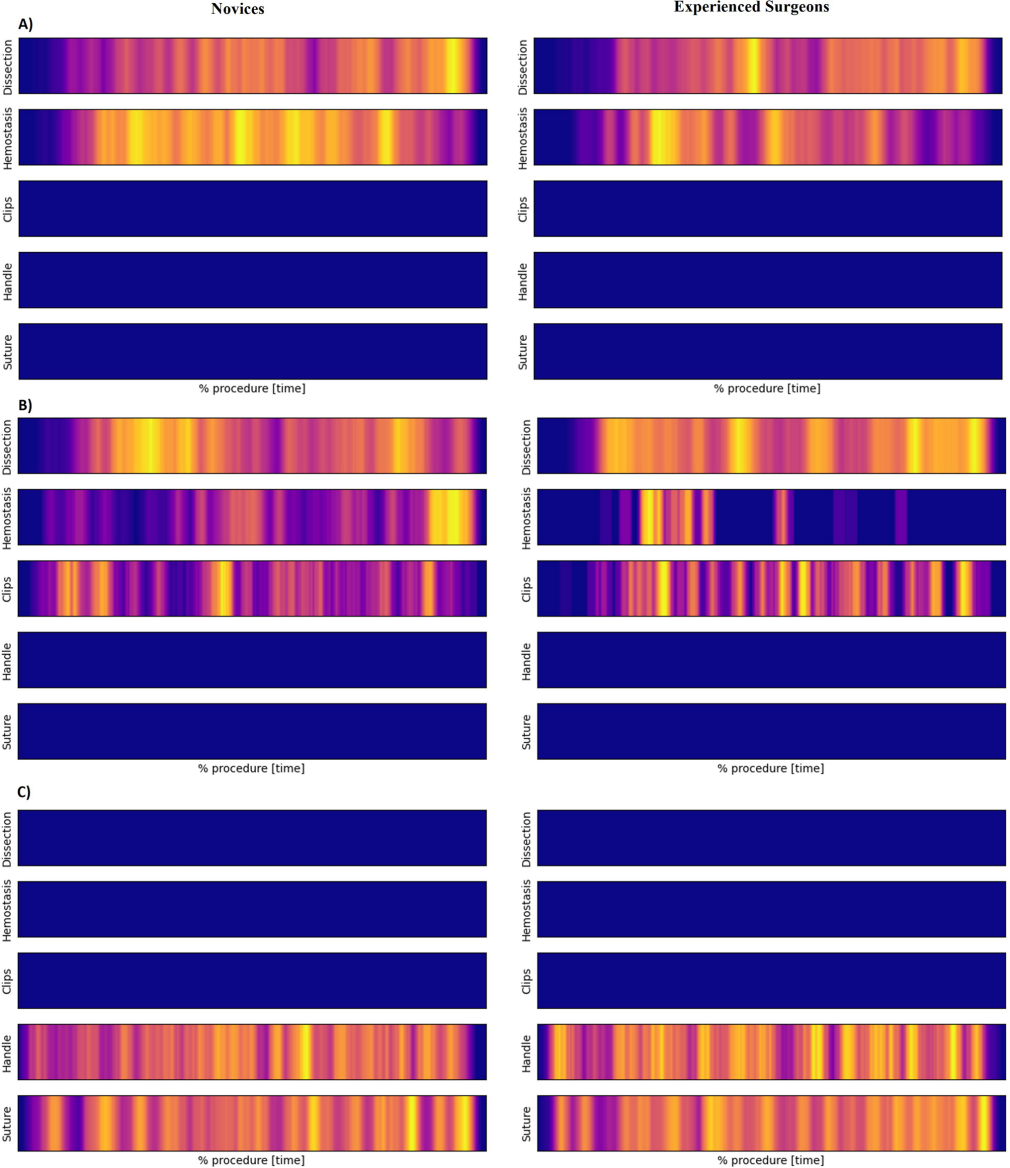
One-dimensional heat maps, *snail tracks*, for the mean distribution of surgical gestures throughout the surgical procedures for experienced surgeons and novices. *Each* *vertical line* of color represents a phase of the corresponding surgical phase (active time). *No vertical color* line is no use of any surgical phases (idle time). The more color, the more activity. (**A**) Bladder-neck dissection, (**B**) Neurovascular-bundle dissection, (**C**) Urethrovesical anastomosis

The average surgical patterns for novices and experienced surgeons are illustrated as *snail tracks* in a one-dimensional heat map (Fig. 3). The *snail tracks* showed the pattern the surgeon performed during the procedure. We saw an overall use of more phases and a different distribution of the phases between novices and experienced surgeons. For each part procedure, there were differences in the patterns between the novices and experienced surgeons. During the bladder-neck dissection, novices tended to use more hemostatic control throughout the procedure, whereas experienced surgeons mostly used this at the beginning of the procedure. This trend was also observed during the neurovascular bundle dissection, where novices used fewer clips and more hemostatic control at the end of the procedure. Further, novices had clusters of regular dissection, whereas experienced surgeons had more consistent use of this gesture throughout the neurovascular bundle dissection. There was no visible difference in the surgical pattern during the urethrovesical anastomosis.

**Fig. 4:**
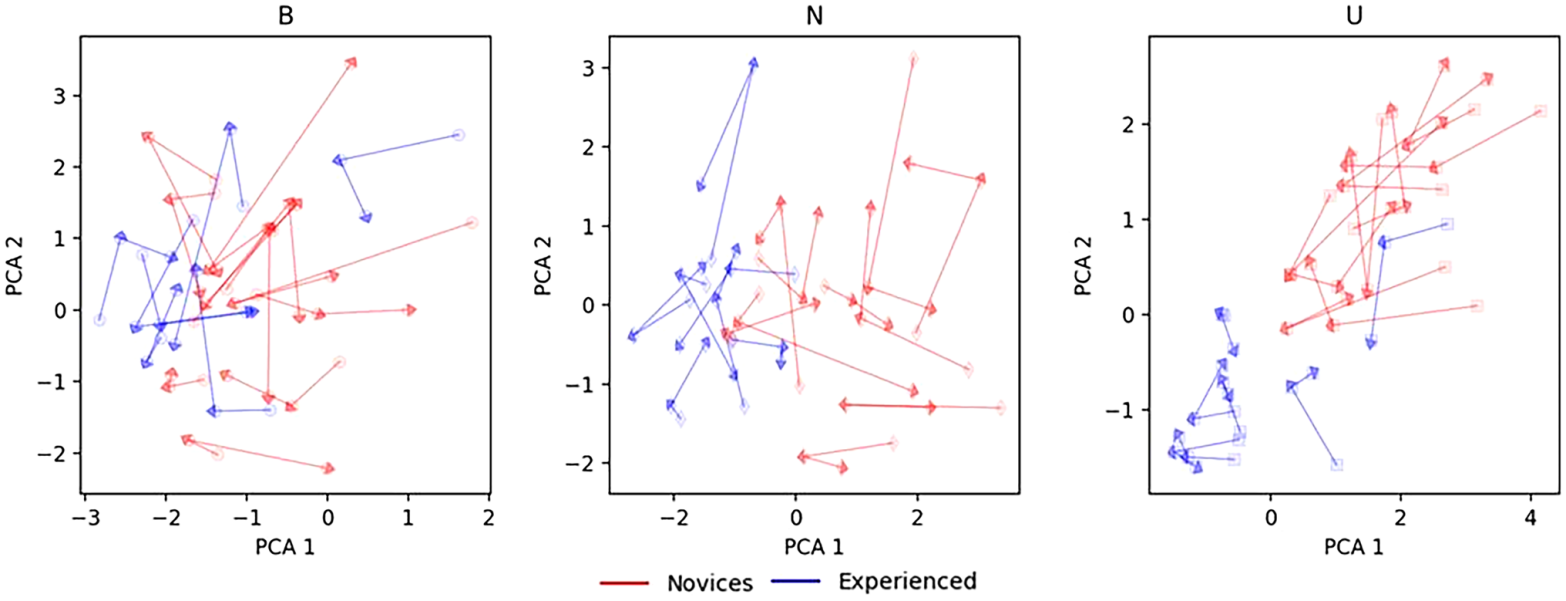
Principal component analysis (PCA). The *arrows* show the distance to the target and the magnitude of the progress for each surgeon. *B: Bladder-neck dissection, N: Neurovascular-bundle dissection, U: Urethrovesical anastomosis*

The PCA showed the performance of the novices and experienced surgeons was grouped when the data set was transformed into principal components (Fig. 4). The arrows show the magnitude of improvement for each surgeon by connecting each surgeon’s attempt. Most surgeons improved and most of the surgeons improved in the same direction. This means the surgeons moved towards the same performance level—the novice surgeons tend to move in the direction of the group of experienced surgeons.

**Fig. 5:**
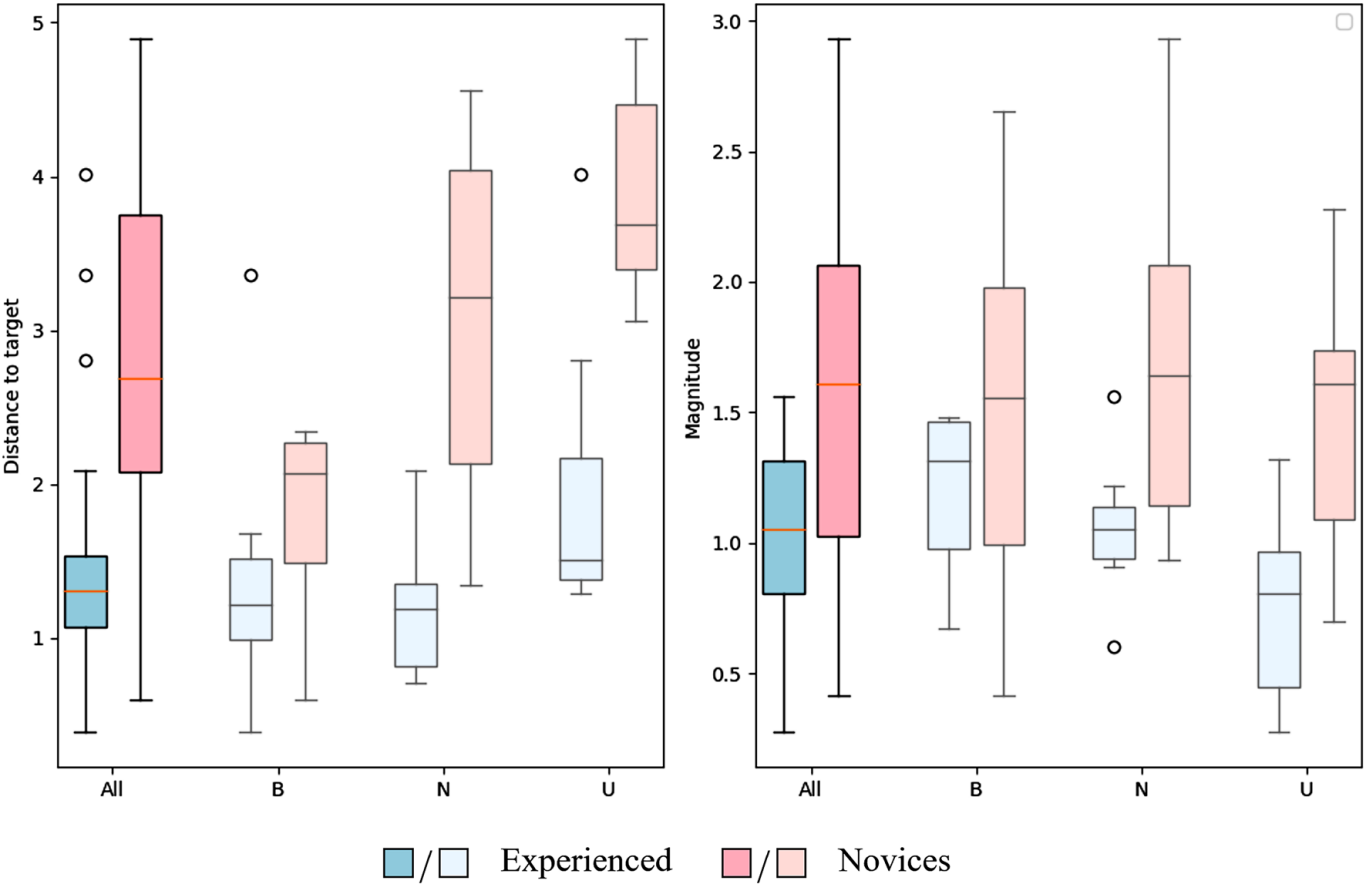
Boxplot showing the distribution and spread in the distance to target and magnitude for the principal component analysis (PCA). *B: Bladder-neck dissection, N: Neurovascular-bundle dissection, U: Urethrovesical anastomosis*

The novices started further away from the performance level than the experienced surgeons (mean distance to target: 2.87 vs. 1.54, *p* < 0.001) (Fig. 5 and Supplementary Table 1) resulting in the novices improving more with each procedure than the experienced surgeons did (mean magnitude: 1.59 vs. 1.00, *p* < 0.001). This did, however, not apply to the bladder-neck dissection where novices and experienced surgeons gained the same amount of improvement with each procedure performed (distance to target *p* = 0.14; magnitude *p* = 0.13).

## Discussion

We have demonstrated that general annotations of surgical gestures can be used to discriminate between different levels of surgical competence in simulated RARP. Overall, novices are more ineffective, spend more time on each part-procedure, and have less economization of their surgical gestures than experienced surgeons. The novices make unnecessary or incorrect dissection gestures, and more hemostatic control which could result in more tissue damage. Surprisingly, the percentage of idle time is similar between the two groups, showing that all surgeons may need to stop, assess the tissue, and plan their next move. These periods of idle time are an unavoidable part of surgery as all surgeons, even experienced surgeons, must adapt to the individual anatomy of the patients and change their surgical plans accordingly [25]. The idle time phases for novices are longer which could indicate that they have more trouble deviating from standard practice than experienced surgeons. As the surgeons become more experienced, they grow more confident, progress to autonomy, and adapt quicker to surgical challenges resulting in shorter periods of idle time [26, 27].

Conventional assessment e.g., automated performance metrics or rater assessment, is only provided on entire procedures e.g., a total time or accumulated performance scores. It does not give details on where in the procedure the training surgeon could improve, only that they could. Consequently, we could risk providing incorrect feedback on the surgical performance of the trainee if we do not focus on procedural progress. Surgical gestures have been proposed to assess the efficacy or experience level of the surgeon and to predict patient outcomes [11, 14, 15]. Using the *snail tracks*, we can provide the novices with valuable feedback on the procedural steps and how they can change their pattern of actions. In our study, there were limited differences in the total time for idle time and active time between novice surgeons and experienced surgeons in bladder-neck dissection. If we look at the *snail tracks*, experienced surgeons use more hemostatic control at the beginning of the procedure, whereas novice surgeons use hemostatic control throughout the procedure. This could indicate that the two groups have different approaches to hemostatic control with the experienced surgeons preventing bleeding early on with the novices stopping bleeding as it occurs.

Surgical gestures have the potential to be used for feedback in both simulated and real-life settings [11, 14, 15]. It could help identify in which areas surgeons could benefit from training or one-to-one feedback, where experienced surgeons can provide more detailed information about how to correctly perform and progress in the procedures. The PCA can be used as a visible feedback tool to show the progress when training. The arrows will show the training surgeon how big the improvement is. If they progress toward the group of experienced surgeons, they are on the track and can use either the total times of the procedures or the *snail tracks* for further feedback on how to improve their surgical competence. If they do not progress or progress in a different direction it could be an indication that they need assistance or other types of feedback to improve. This should be used for feedback post-procedure to not disturb or increase the cognitive load of the training surgeon. The use of PCA and *snail tracks* as feedback tools has not been proposed before. These tools could be a well-needed solution in addition to the presence of a senior surgeon to provide feedback. They could be both cost-effective and time-saving so that the senior surgeons could use their resources on the trainees who need one-on-one feedback.

Limitations of this study include a low number of experienced RARP surgeons, which could induce a distorted pattern of surgical gestures. The surgeons had performed between 70 and 800 procedures at the time of inclusion, but the learning curve for RARP can vary between 20 and >700 procedures [28]. In a previous validity study, we found that the simulator metrics could discriminate between the skill level of the novices and experienced RARP surgeons, even with the varying experience in the experienced RARP group. The reason for this is most likely because the simulator exercises are standardized without the anatomical variations usually seen in real-life cases. The level of experience of the surgeons to perform RARP may be different in a simulated environment than in real life. Therefore, it may not be necessary to be an expert to perform the simulated RARP well. Further, we only analyzed simulated procedures instead of real-life surgeries but we chose this as the simulated environment allows the independent performance of novices even in their early learning periods without jeopardizing patient safety. Furthermore, virtually simulated procedures are all on the same patient case, minimizing case-by-case variation [29]. This method was tested in a simulated environment and more research is required to prove that the same approach could be used for assessment of real-life surgeries and other procedures.

With the introduction of robotic surgery, recording videos of surgical procedures has become easier. The amount of data that can potentially be extracted from the videos of the robotic procedures can be used to recognize and analyze the performance of the surgeon [17, 30–32]. These analyses can be used to create AI algorithms to help predict patient short- and long-term outcomes [11, 12, 14, 15, 33]. Compared to prediction models based on pre-operative patient characteristics alone, surgical gestures take the performance of the surgeon into account together with the complexity of the surgery. Surgical gestures are a better predictor of surgical outcomes compared to traditional patient characteristics such as BMI, age, and biopsy results before surgery [11, 12, 14, 15, 33].

Unfortunately, video data processing is an essential step for the recognition of surgical gestures, which currently requires manual annotation of video data which is very time-consuming [3, 7, 15, 18]. Technological advances such as machine learning have the potential for the automatic recognition of surgical gestures. This can potentially deliver immediate individual feedback, increasing the efficiency of surgical training, and helping the early detection of areas that need improvement. However, these machine-learning models require a large amount of data and are still not generalizable. One major challenge in the use of surgical gestures for performance evaluation is that there is currently no consensus on how gestures are defined and annotated. This makes sharing datasets and AI algorithms difficult [4, 7, 15, 17, 18]. Various approaches with varying levels of data granularity have previously been used with success. We used a general classification of surgical gestures to assess surgical skills whereas former studies have deconstructed each surgical gesture into even smaller parts [11, 14–18]. Our approach could potentially reduce the data load and allow for simpler, more feasible AI algorithms and thereby faster implementation.

## Conclusion

General surgical gestures can distinguish between surgical competence in simulated RARP and be displayed as a visual tool to show how performance is improving. The pass/fail level can be used to ensure the competence of the training surgeon before proceeding with supervised real-life surgery. The next step is to investigate if the developed tool can optimize automated feedback during simulator training.

## Supplementary Information

Below is the link to the electronic supplementary material.

Supplementary file1 Independent t-tests between novice surgeons and experienced surgeons for the distance to target and magnitude for the principal component analysis (PCA) (DOCX 14 KB)

Supplementary file2 Examples of the general surgical gestures used for annotation: regular dissection, hemostatic control, application of clips, needle handling, and suturing (AVI 18282 KB)

## Data Availability

Data available on request from authors and participant consent.
