# Supplementary material for: Surgical gestures can be used to assess surgical competence in robot-assisted surgery: A validity investigating study of simulated RARP
Source: J Robot Surg. 2024 Jan 20;18(1):47. doi: 10.1007/s11701-023-01807-4 (PMC10799775; doi:10.1007/s11701-023-01807-4)
Supplement: Supplementary file 1 — Supplementary file1 Independent t-tests between novice surgeons and experienced surgeons for the distance to target and magnitude for the principal component analysis (PCA) (DOCX 14 KB) [file 11701_2023_1807_MOESM1_ESM.docx]

|  | **Distance to target** *Mean (SD)* | | | **Magnitude** *Mean (SD)* | | |
| --- | --- | --- | --- | --- | --- | --- |
|  | Novice | Experienced | p-value | Novice | Experienced | p-value |
| **Total procedure** | 2.87 (1.2) | 1.54 (0.9) | <0.001* | 1.59 (0.6) | 1.00 (0.4) | <0.001* |
| **Bladder-neck dissection** | 1.81 (0.5) | 1.42 (0.9) | 0.14 | 1.53 (0.7) | 1.19 (0.3) | 0.13 |
| **Neurovascular-bundle dissection** | 3.10 (1.1) | 1.20 (0.4) | <0.001* | 1.71 (0.6) | 1.05 (0.23) | 0.009* |
| **Urethrovesical anastomosis** | 3.90 (0.6) | 1.99 (1.0) | 0.001* | 1.52 (0.5) | 0.75 (0.4) | 0.010* |
| **Significance level of p<0.05* |  |  |  |  |  |  |
